# Cardiovocal Syndrome Associated With Idiopathic Pulmonary Arterial Hypertension: A Case Report and Literature Review

**DOI:** 10.7759/cureus.27070

**Published:** 2022-07-20

**Authors:** Kohhei Ohi, Jun Suzuki, Ryoukichi Ikeda, Risako Kakuta, Yukio Katori

**Affiliations:** 1 Department of Otolaryngology-Head and Neck Surgery, Tohoku University Graduate School of Medicine, Sendai, JPN

**Keywords:** recurrent laryngeal nerve palsy, hoarseness, ortner syndrome, idiopathic pulmonary arterial hypertension, cardiovocal syndrome

## Abstract

Cardiovocal syndrome is left recurrent laryngeal nerve palsy associated with cardiovascular disease. Herein, we report a rare case of left recurrent laryngeal nerve palsy caused by idiopathic pulmonary arterial hypertension. A 40-year-old woman diagnosed with idiopathic pulmonary arterial hypertension was referred to our department for occult infection foci in the ear, nose, and throat (ENT). She had no apparent subjective symptoms in the ENT area, including hoarseness. Flexible laryngoscopy revealed left vocal cord paralysis, and contrast-enhanced computed tomography revealed dilatation of the pulmonary trunk, bilateral pulmonary arteries, and right ventricle, suggesting compression of the left recurrent laryngeal nerve. In our daily practice, when we encounter a left recurrent laryngeal nerve palsy that seems to be endogenous, cardiovascular lesions should be ruled out.

## Introduction

Cardiovocal syndrome, also known as Ortner’s syndrome, is a general term for left recurrent laryngeal nerve palsy associated with cardiovascular disease. Ortner first reported left recurrent laryngeal nerve palsy due to mitral stenosis in 1897 [1]. It has also been reported to be associated with various cardiovascular diseases apart from mitral stenosis. Idiopathic pulmonary arterial hypertension (IPAH) is a rare disease estimated to occur in approximately 50 cases per million people; however, there is a limited number of cardiovocal syndrome cases reported to be associated with IPAH [2-14]. IPAH is a progressive disease that affects pre-pulmonary vessels and is characterized by elevated pulmonary artery (PA) pressure with no apparent cause and a high mortality rate. Here, we report an uncommon case of cardiovocal syndrome associated with IPAH, review previous cases, and discuss the characteristics of cardiovocal syndrome associated with IPAH.

## Case presentation

**Figure 1:**
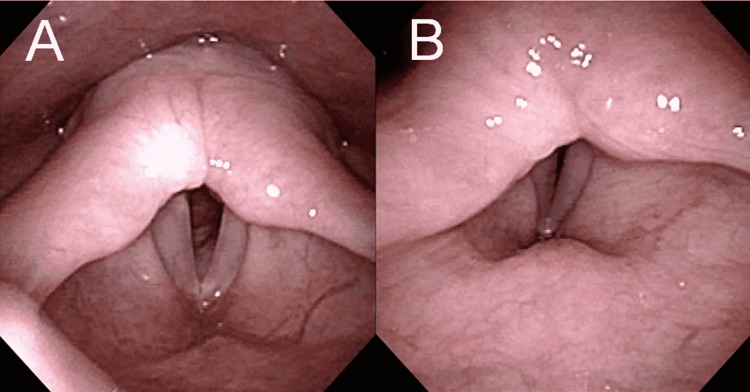
Flexible laryngoscopic images showing immobility of the left vocal fold A finding during (A) resting breathing and (B) vocalization.

**Figure 2:**
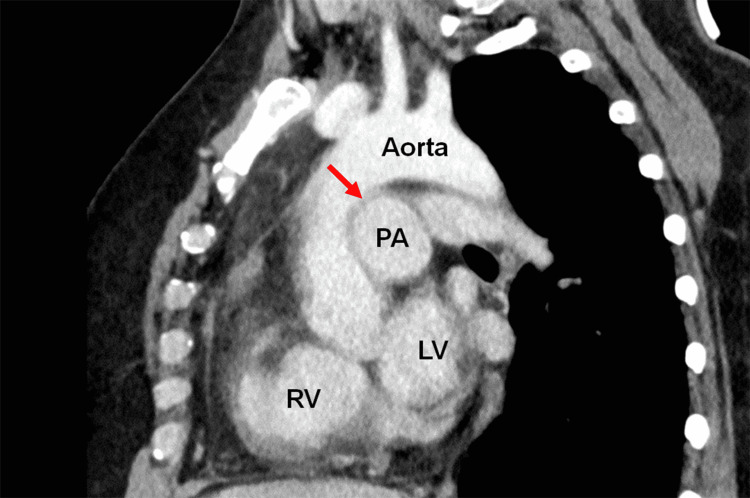
Computed tomography (CT) scan of the chest The sagittal CT image shows dilation of the main pulmonary artery and enlargement of the right ventricle, consistent with typical idiopathic pulmonary arterial hypertension findings. Dilation of the pulmonary artery results in the narrowing of the space between the pulmonary artery and aorta (red arrow) and causes compression of the recurrent laryngeal nerve. LV: left ventricle; PA: pulmonary artery; RV: right ventricle

A 40-year-old woman diagnosed with IPAH was referred to our department for occult infection foci in the ear, nose, and throat (ENT) before lung transplantation. She had no apparent subjective symptoms in ENT areas, like hoarseness, at presentation. At the age of 33 years, she developed shortness of breath on exertion. She was diagnosed with IPAH two years after symptom onset, and her initial right heart catheterization documented increased pulmonary arterial pressure (PAP) of 110/38 (mean 67) mmHg. Although she was regularly followed up and treated with medications such as treprostinil, epoprostenol, macitentan, and selexipag, severe diarrhea and septic shock occurred repeatedly, and pulmonary hypertension gradually progressed. Finally, lung transplantation was determined to be indicated due to physical limitations. She had no history of smoking or drinking alcohol, tumor lesions, or neck or chest surgery. There were no palpable cervical lymphadenopathy, mass lesions, or enlarged thyroid gland. Flexible laryngoscopy revealed left true vocal fold palsy (Figure 1), and contrast-enhanced computed tomography (CT) showed dilatation of the pulmonary trunk, bilateral pulmonary arteries, and the right ventricle (Figure 2).

There were no apparent mass lesions in the skull base, neck, thyroid gland, or chest region. The space between the pulmonary arteries and the aortic arch was narrowed because of dilation of the pulmonary arteries, suggesting compression of the left recurrent laryngeal nerve. Although mild hoarseness with shortened maximum phonation time (10 s) was observed, the patient did not wish to receive treatment for speech improvement. She was followed up as an outpatient without obvious subjective symptoms. Written informed consent was obtained from the patient.

## Discussion

Herein, we report a rare case of cardiovocal syndrome associated with IPAH. In recurrent laryngeal nerve palsy, tumor lesions in cervicothoracic malignancies and aortic aneurysms are critical and life-threatening diseases, and CT is useful for identifying these lesions [15]. Previous reports have shown that the rate of cardiovocal syndrome in patients with recurrent laryngeal nerve palsy is approximately 1% [16,17]. Various cardiovascular diseases cause cardiovocal syndrome, but only a few reported cases of cardiovocal syndrome are associated with IPAH. Most nonsurgical recurrent laryngeal nerve palsies are idiopathic or caused by lung and neck tumors or aortic aneurysms. However, cardiovascular diseases, including IPAH, can cause recurrent laryngeal nerve palsy and should be diagnosed with caution.

Past cases reported as cardiovocal syndrome caused by IPAH and the present case are summarized in Table 1; in total, 23 patients were identified, with age and sex confirmed in 13 cases [3-14].

**Table 1:** Summary of 23 cases of cardiovocal syndrome caused by idiopathic pulmonary arterial hypertension. IPHA: idiopathic pulmonary arterial hypertension; PA: pulmonary artery; mPAP: mean pulmonary artery pressure; M: male; F: female; N/A: not available

Author, Year, Reference	Number	Age	Sex	Hoarseness at presentation	Time from IPAH symptoms (months)	Size of PA (cm)	mPAP (mmHg)	Improvement of hoarseness after IPAH treatments
Brinton, 1950 [3]	1	26	M	No	N/A	N/A	N/A	No
Soothill, 1951 [4]	1	22	M	Yes	9	N/A	98	No
Kagal et al., 1975 [5]	2	28, 25	1M, 1F	Yes in 1	2.4	N/A	N/A	No
Shah and Shah, 1975 [6]	10	N/A	N/A	Yes in 6	N/A	N/A	N/A	No
Wilmshurst et al., 1983 [7]	1	37	M	Yes	4	N/A	57	No
Sengupta et al., 1998 [8]	1	37	M	Yes	8	2.3	62	No
Rajasekhar et al., 2014 [9]	1	35	F	Yes	Simultaneously	N/A	N/A	Yes
Shankar and Lohiya, 2014 [10]	1	19	M	Yes	11	3.8	101	Yes
Dakkak and Tonelli, 2016 [11]	1	42	F	Yes	N/A	3.5	45	N/A
Garg et al., 2017 [12]	1	23	F	Yes	2	N/A	50	Yes
Kardos et al., 2017 [13]	1	18	F	Yes	3	4.59	N/A	N/A
Jalil et al., 2019 [14]	1	34	F	Yes	36	4.1	81	Yes
Present case	1	40	F	Yes	84	4.2	67	No

Six of the 13 patients were male, and there was no difference in the ratio of males to females. Five out of the 13 patients were in their 20s, and four were in their 30s, indicating a high ratio of young to middle-aged patients. There were no cases in the ≥ 50 years age group. The mean diameter of the main PA was reported as 2.72 cm in healthy subjects [18]. In our review of six cases where numbers were available, the mean diameter of the main PA was 3.75 ± 0.80 cm. PA pressure was noted in eight patients. Seventeen of the 23 patients (73.9%) showed subjective hoarseness, and in four cases (17.4%), hoarseness was improved by specific treatment for IPAH. IPAH is a rare but important cause of left recurrent laryngeal nerve palsy in young patients, and confirming the presence of a dilated PA is essential for diagnosis. It is also noteworthy that palsy may improve with appropriate treatment, and that approximately 1/4 of cases of vocal cord paralysis in IPAH did not show hoarseness as a subjective symptom.

## Conclusions

In conclusion, it is necessary to consider cardiovascular diseases such as IPAH as a cause of left recurrent laryngeal nerve palsy. Based on a literature review of cardiovocal syndrome caused by IPAH, appropriate interventions for IPAH may effectively treat hoarseness caused by left recurrent laryngeal nerve palsy.
